# Inhibition of UDP/P2Y_6_ purinergic signaling prevents phagocytosis of viable neurons by activated microglia *in vitro* and *in vivo*

**DOI:** 10.1002/glia.22693

**Published:** 2014-05-19

**Authors:** Jonas J Neher, Urte Neniskyte, Tamara Hornik, Guy C Brown

**Affiliations:** Department of Biochemistry, University of CambridgeCambridge, United Kingdom

**Keywords:** amyloid β, neuroinflammation, P2Y_6_ receptor, uridine diphosphate, phagoptosis

## Abstract

Microglia activated through Toll-like receptor (TLR)-2 or -4 can cause neuronal death by phagocytosing otherwise-viable neurons—a form of cell death called “phagoptosis.” UDP release from neurons has been shown to provoke microglial phagocytosis of neurons via microglial P2Y_6_ receptors, but whether inhibition of this process affects neuronal survival is unknown. We tested here whether inhibition of P2Y_6_ signaling could prevent neuronal death in inflammatory conditions, and whether UDP signaling can induce phagoptosis of stressed but viable neurons. We find that delayed neuronal loss and death in mixed neuronal/glial cultures induced by the TLR ligands lipopolysaccharide (LPS) or lipoteichoic acid was prevented by: apyrase (to degrade nucleotides), Reactive Blue 2 (to inhibit purinergic signaling), or MRS2578 (to specifically block P2Y_6_ receptors). In each case, inflammatory activation of microglia was not affected, and the rescued neurons remained viable for at least 7 days. Blocking P2Y_6_ receptors with MRS2578 also prevented phagoptosis of neurons induced by 250 nM amyloid beta 1–42, 5 μM peroxynitrite, or 50 μM 3-morpholinosydnonimine (which releases reactive oxygen and nitrogen species). Furthermore, the P2Y_6_ receptor agonist UDP by itself was sufficient to stimulate microglial phagocytosis and to induce rapid neuronal loss that was prevented by eliminating microglia or inhibiting phagocytosis. *In vivo*, injection of LPS into rat striatum induced microglial activation and delayed neuronal loss and blocking P2Y_6_ receptors with MRS2578 prevented this neuronal loss. Thus, blocking UDP/P2Y_6_ signaling is sufficient to prevent neuronal loss and death induced by a wide range of stimuli that activate microglial phagocytosis of neurons.

## Introduction

We have previously shown that activated microglia can cause neuronal death by phagocytosing otherwise-viable neurons—a form of cell death called “primary phagocytosis” or “phagoptosis” (Brown and Neher, 2014). *In vitro* we have shown that this phagocytosis-induced death is mediated by peroxynitrite released from inflammatory activated microglia, inducing reversible exposure of the “eat-me” signal phosphatidylserine (PS) on the surface of viable neurons, which provokes microglial phagocytosis of such neurons via the PS-binding protein MFG-E8 and its microglial vitronectin receptor (VNR) (Fricker et al., 2012a; Neher et al., 2011b; Neniskyte and Brown, 2013; Neniskyte et al., 2011). Importantly, a similar mechanism appears to cause neuronal loss during inflammation *in vivo* (Fricker et al., 2012a; Neher et al., 2013).

Phagoptosis is probably the most common form of cell death in the body physiologically (Brown and Neher, [Bibr b7],[Bibr b8]). With regard to pathology, we have shown that primary phagocytosis of neurons can be induced by nanomolar levels of the Alzheimer's disease-related peptide amyloid beta (Aβ), or by the Toll-like receptor (TLR)2 activator lipoteichoic acid (LTA) or by the TLR4 activator lipopolysaccharide (LPS) and that phagocytosis of viable neurons mediates delayed neuronal loss after focal brain ischemia (Fricker et al., 2012a; Neher et al., [Bibr b28],[Bibr b26]; Neniskyte and Brown, 2013; Neniskyte et al., 2011). Others have recently shown that primary phagocytosis of otherwise viable neurons by activated microglia mediates neuronal loss and death in models of AIDS dementia, Parkinson's disease, Motor Neuron Disease, and developmental loss of neuronal precursors that may be related to Autism or Schizophrenia (Barcia et al., 2012; Cunningham et al., 2013; Liu et al., 2012; Marker et al., 2012). Thus, it is important to understand the signaling between neurons and microglia that drives phagoptosis, and thereby devise therapies that prevent it in neurological diseases.

Phagocytosis of cells normally requires activation of several different signaling pathways including the exposure of “eat-me” signals on the target cell and their detection by soluble bridging molecules or cell surface receptors on the phagocyte (Ravichandran, 2011). Fairly recently, it was found that in a final step microglial phagocytosis of neurons also requires the release of UDP from the target cell, potentially inducing a switch from a migratory to a phagocytic phenotype (Bernier et al., 2013) and leading to formation of the so-called phagocytic cup (the membrane invagination forming around the target cell) and thereby inducing uptake of the target cell (Koizumi et al., 2007). In kainate-treated animals, it was shown that phagocytosis required UDP release from neurons triggering formation of the phagocytic cup in adjacent microglia via purinergic P2Y_6_ receptors (Koizumi et al., 2007). However, whether inhibition of P2Y_6_ signaling would affect neuronal survival was not investigated, because it was assumed by the authors that microglia only phagocytose dead or dying neurons. Thus, we were interested in whether signaling between stressed neurons and activated microglia via UDP/P2Y_6_ might also mediate phagoptosis of otherwise-viable neurons.

We show here that UDP/P2Y_6_ signaling does indeed mediate phagoptosis of neurons by microglia activated by a wide range of inflammatory stimuli, and that blocking P2Y_6_ receptors is sufficient to prevent neuronal loss and death in culture and *in vivo*. Thus, P2Y_6_ may be an attractive target for drug development to prevent inflammatory neuronal loss in a wide range of neurological diseases.

## Materials and Methods

### Reagents

Cell culture reagents were from PAA. Purified LTA was from Invivogen; LPS, apyrase and reactive blue 2 (RB2), were from Sigma, authentic peroxynitrite was from Cayman, 3-morpholinosydnonimine (SIN-1) was from Invitrogen, MRS2578, MRS2693, and UDP were from Tocris, Amyloid β_1-42_ peptide was from EZBiolab, Annexin V was from BioVision. Tumor necrosis factor-α and interleukin-1β Quantikine Elisa Kits were from R&D Systems. NeuN antibody was from Millipore, β-tubulin III antibody was from Sigma, anti-cleaved caspase-3 antibody was from Cell Signaling Technology, Alexa Fluor 488-labeled *Griffonia simplicifolia* isolectin B_4_ and secondary goat-anti-mouse-Alexa Fluor 488 antibody were from Invitrogen, secondary goat anti-mouse-Cy3 and goat anti-rabbit-Cy3 antibodies were from Jackson ImmunoResearch Laboratories. All other materials were purchased from Sigma.

### Preparation of Aβ Solution

Amyloid beta 1–42 (Aβ_1–42_) peptide was dissolved in 1,1,1,3,3,3-hexafluorisopropanol (HFIP) at 2.5 mg/mL concentration and incubated for 30–60 min at room temperature. HFIP was removed by evaporation and Aβ was resuspended in dimethyl sulfoxide at 5 mM concentration. Before adding to the cultures, Aβ was prediluted in culture medium to 25 μM concentration.

### Primary Cell Culture

All experiments were performed in accordance with the UK Animals (Scientific Procedures) Act (1986) and approved by the Cambridge University local ethical committee. Primary mixed neuronal/glial cultures were prepared from cerebella of postnatal day 5–7 Wistar rat pups as previously described (Neher et al., 2011a) and were plated on poly-l-lysine coated plates and allowed to mature *in vitro* for 7–9 days before treatment. At this point, the culture composition of these “mixed cultures” was 85 ± 5% neurons, 7 ± 3% astrocytes, and 5 ± 3% microglia (Bal-Price and Brown, 2001). When desired, microglia were selectively eliminated after 6 DIV by treatment with l-leucine-methyl-ester (LME, 50 mM) for 4 h and medium was replaced with conditioned medium from sister cultures (these cultures will be referred to as “microglia-depleted” and contained 0.7 ± 0.2% microglia; Neher et al., 2011b).

### Cell Treatment

Cells were treated with 50 μg/mL of LTA, 100 ng/mL of LPS, 250 nM of amyloid β_1-42_, 5 μM of peroxynitrite, or 50 μM of SIN-1 for 3–7 days; 100 μM UDP was added for 6, 24, or 72 h, MRS2693 was added for 24 or 72 h. RB2 (100 μM) was added together with LTA or LPS, MRS2578 (1 μM) was added every day, apyrase (1 U/mL) was added 2 days after culture stimulation.

### Quantification of Cell Numbers *In Vitro*

Cell densities were evaluated as previously described (Neher et al., 2011b). In brief, cell densities were assessed in live cultures after staining with the nuclear dyes Hoechst 33342 (5 μg/mL) and propidium iodide (1 μg/mL); Alexa-488–tagged isolectin-B4 (1 μg/mL) was used to identify microglia. Healthy and apoptotic (chromatin-condensed) neurons were recognized by their distinct nuclear morphology, whereas propidium-iodide positive cells (indicating membrane permeabilization) were scored as necrotic. Four microscopic fields/well (between 150 and 200 neurons per field in control wells) in 2 wells/condition were quantified for a single experiment. Experiments performed with cultures from at least three independent culture preparations were used for statistical analysis. For tetramethylrhodamine methyl ester (TMRM) staining, cells were incubated with TMRM (3 nM) for 30 min at 37°C, 5% CO_2_. Cell nuclei were counterstained with Hoechst 33342 (10 μg/mL). Total cell densities and TMRM-positive cell numbers were evaluated using a Leica DMI6000 CS microscope.

### Phagocytosis Assay

Cultures were incubated with a 0.0012% w/v solution of 1 μm carboxylate-modified microspheres (Invitrogen) or a 0.0015% w/v solution of 5–5.9 μm carboxyl fluorescent Nile red particles (Spherotech) for 2 h. The medium was removed, and cells washed with ice-cold PBS to remove unattached beads. Cells were stained with Hoechst (4 μg/mL) and Alexa 488-conjugated *Griffonia simplicifolia* isolectin-B4 (1 μg/mL), imaged using a Leica DMI6000 microscope and the number of beads per cell counted. Four microscopic fields (each 1.9 × 10^5^ μm^2^) per well in four wells per condition were quantified for a single experiment.

### Assessment of Inflammatory Mediators

Tumor necrosis factor-α and interleukin-1β levels were determined using rat Quantikine Elisa Kits (R&D Systems) by following the manufacturer's protocols. Nitric oxide (NO) release was evaluated by assessing nitrite levels with the Griess reaction as described previously (Kinsner et al., 2005).

### Immunocytochemistry

For immunofluorescent labeling, cultures were grown on poly-l-lysine-coated glass coverslips. At the end of the treatment, cultures were fixed with 4% paraformaldehyde (PFA, in PBS, pH 7.4), blocked with 10% normal serum of the secondary antibody host-species in PBS containing 0.3% Triton-X 100, and incubated with the primary anti-NeuN (Chemicon, 1:100), anti-cleaved caspase-3 (Cell Signaling Technology, 1:250) antibodies overnight at 4°C. Then, cells were incubated with the secondary antibody in PBS containing 4% normal serum and 0.3% Triton-X 100 for 1 h at room temperature. Coverslips were mounted using Vectashield mounting medium (Vector Laboratories) and analyzed by confocal microscopy.

### Surgical Procedure

Ten-week-old male Wistar rats were used for bilateral striatal stereotactic injections. Animals were maintained under anesthesia with isoflurane throughout, mounted in a stereotactic frame (Stoelting) and injected at the following coordinates relative to bregma: anterior-posterior +1.0, medio-lateral ±2.6, and dorso-ventral −5.0 mm. Rats received bilateral injections (2 μL) of either (1) Hank's Buffered Salt Solution (HBSS) and LPS (5 μg), or (2) MRS2578 (MRS, 100 μM) and MRS + LPS. Infusion speed was 24 μL/h, using a 31G stainless steel needle, which was left in place for 2 min after injection to prevent back-flow. After 3 days, animals were deeply anesthetized with pentobarbitol and transcardially perfused with cold PBS (0.01 M, pH 7.4), followed by 4% PFA (0.1 M phosphate buffer [PB], pH 7.4). Brains were kept in PFA for 24 h, then in 30% sucrose solution (0.1 M PB, pH 7.4) for at least 3 days, and cut into 40-μm-thick coronal sections on a sliding microtome (Leica).

### Immunohistochemistry

Sections were incubated for 2 h, RT, in PBS plus 5% normal serum of the secondary antibody species and 0.3% Triton X-100 (all Sigma). Sections were incubated with primary antibodies overnight, 4°C (for control staining the antibody was omitted), washed, and incubated with the secondary antibody for 2 h, 4°C. Sections were washed, mounted, and coverslipped using FluorSave reagent. Antibodies used were anti-NeuN (Chemicon, 1:500), anti-β-tubulin III (Sigma, 1:500), and IB4 (1 μg/mL, Invitrogen). Secondary antibodies were from Jackson Immunolaboratories (1:200). For TUNEL/NeuN/IB4 labeling, sections were stained using a TUNEL kit (Roche), then with anti-NeuN antibody and biotinylated isolectin-B4 (Sigma, 1:200; 48 h, 4°C). Sections were washed, incubated with donkey anti-mouse-Cy3 antibody (Jackson ImmunoResearch, 1:100), washed, and treated with Streptavidin-Alexa647 (Molecular Probes, 1:500), 2 h each, RT. Sections were analyzed on an Olympus Fluoview 300 confocal microscope.

### Quantification of Neuronal Densities *In Vivo*

Five coronal sections were analyzed per animal at coordinates AP+0.6, +0.8, +1.0, +1.2, and +1.4 mm identified according to Paxinos and Watson (1982). At least five microscopic fields (20×) were analyzed per section that showed microglial activation (these never contained the needle tract). Neuronal densities were determined for 3 day time-points using NeuN and β-tubulin III staining. NeuN-positive nuclei were automatically quantified using Image J software using the “analyze particles” function and defining a threshold and particle size/circularity that enforced recognition of nuclei located completely within the section and focal plane (this was manually validated first). β-Tubulin III positive cells were counted manually by a blinded observer, excluding cells that did not show a complete cytoplasmic ring of β-tubulin staining, which were considered to be situated outside of the section.

### Statistical Analysis

Statistical analysis was performed using SPSS software. Normality of data was verified using Shapiro-Wilk test. *In vitro* data were analyzed using one-way ANOVA and *post hoc* Bonferroni test. *In vivo* data were analyzed based on raw data using ANOVA and *post hoc* Sidak test, but are presented as normalized data for ease of comparison. Ipsilateral/contralateral counts were specified as resulting from the same animal (ANCOVA for condition with animal ID as covariate). Results were considered significant if *P* < 0.05.

## Results

### Inhibition of Purinergic Signaling Prevents Inflammatory Neurodegeneration Induced by LTA or LPS

**Figure 1:**
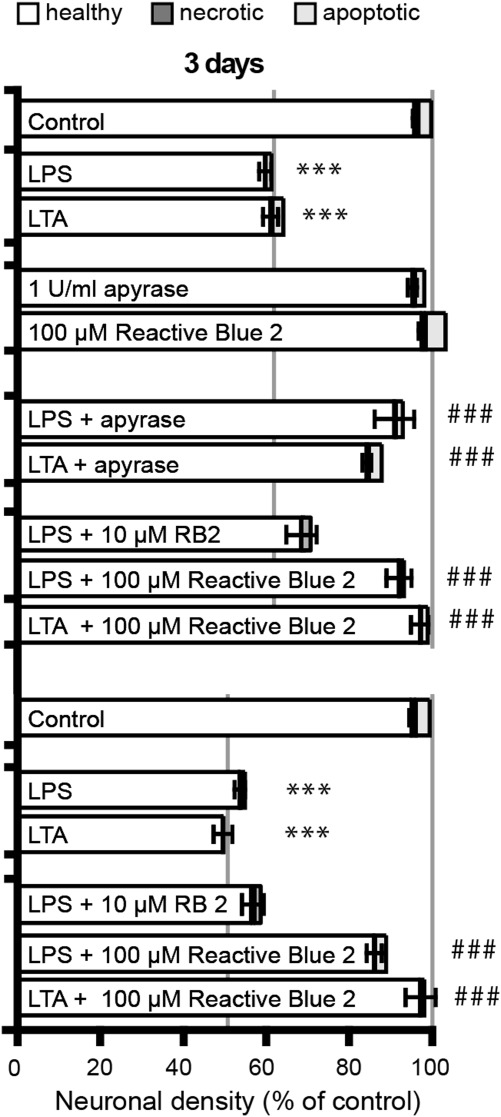
Inhibition of purinergic signaling prevents inflammatory neurodegeneration induced by LTA or LPS. Degradation of di- and tri-phosphate nucleotides by apyrase (1 U/mL, added 2 days after stimulation) inhibits neuronal loss in response to lipoteichoic acid (LTA, 50 μg/mL) or lipopolysaccharide (LPS, 100 ng/mL). The P2X/Y-receptor antagonist Reactive Blue 2 (RB2, 100 μM) efficiently prevents neurodegeneration induced by LTA or LPS as evaluated after 3 and 7 days. Note that apyrase and RB2 did not affect microglial activation by LTA or LPS as measured by proliferation (see Table1). Data are presented as means ± s.e.m. for at least three independent experiments; **/***P < 0.01/0.001 versus control, ##/###*P* < 0.01/0.001 versus LTA or LPS.

We have previously shown that in mixed neuronal/glial cultures (consisting on average of 85 ± 5% neurons, 7 ± 3% astrocytes, and 5 ± 3% microglia) the TLR2 ligand LTA or the TLR4 ligand LPS lead to microglia-dependent neuronal loss that is mediated by microglial phagocytosis of stressed but viable neurons (Fricker et al., 2012a; Neher et al., 2011b). To assess the contribution of purinergic signaling to microglial phagocytosis of inflammatory stressed neurons, LTA- or LPS-stimulated mixed neuronal/glial cultures were treated with the enzyme apyrase (grade III). Apyrase hydrolyses tri- and di-phosphate nucleotides (Kurpius et al., 2007; Warny et al., 2001) and should therefore abrogate both ATP- and UDP-signaling. Because ATP release is also known to be involved in early inflammatory responses to LPS stimulation (such as IL-1β release; Bianco et al., 2005; Inoue, 2002), and because we have reported previously that the majority of neurons are lost between 2 and 3 days after LPS- or LTA-treatment (Fricker et al., 2012a; Neher et al., 2011b; Neniskyte et al., 2011), apyrase was added to mixed cultures 2 days after LPS- or LTA-treatment. Apyrase efficiently inhibited neuronal loss measured at 3 days after stimulation with either LTA or LPS, without affecting the number of necrotic or apoptotic neurons (Fig. 1). Importantly, LTA and LPS caused microglial proliferation in the cultures, an indication of microglial activation (Kinsner et al., 2006; Mander et al., 2006), and this was not inhibited by apyrase (Table1), consistent with delayed apyrase treatment not affecting the inflammatory activation of microglia. These data indicate that purinergic nucleotides were involved in mediating inflammatory neurodegeneration stimulated by LTA or LPS.

**Table 1:** Inhibition of UDP/P2Y_6_ Signaling Does Not Affect Microglial Inflammation

A. Inflammatory responses to LPS and LTA ± inhibitors					
Inhibitor	Measure	Experimental conditions			
Apyrase		Control	LTA	Apyrase	LTA + apyrase
	% Microglia	9.9 ± 1.6	17.9 ± 3.2	9.9 ± 1.5	15.5 ± 1.7
		Control	LPS	Apyrase	LPS + apyrase
	% Microglia	10.2 ± 0.7	20.5 ± 0.9^*^^*^	12.3 ± 1.1	19.8 ± 0.5
Reactive Blue 2 (RB2) 3 days		Control	LTA	100 μM RB2	LTA + 100 μM RB2
	% Microglia	8.6 ± 0.5	17.9 ± 0.9^*^^*^^*^	8.4 ± 0.5	18.4 ± 0.9
		Control	LPS		LPS + 100 μM RB2
	% Microglia	10.6 ± 0.6	20.6 ± 0.2^*^^*^^*^		20.3 ± 0.6
Reactive Blue 2 (RB2) 7 days		Control	LTA	100 μM RB2	LTA + 100 μM RB2
	% Microglia	9.9 ± 1.3	37.4 ± 5.3^*^^*^	11.0 ± 1.2	36.3 ± 4.7
		Control	LPS		LPS + 100 μM RB2
	% Microglia	8.9 ± 0.6	30.5 ± 7.4		32.4 ± 8.5
MRS2578 3 days		Control	LTA	1 μM MRS	LTA + 1 μM MRS
	% Microglia	8.3 ± 0.9	17.0 ± 1.8^*^	9.0 ± 1.1	15.3 ± 1.9
	TNFα [pg/mL]	u.d.	454.6 ± 113.1^*^	u.d.	314.6 ± 72.2
	IL-1β [pg/mL]	u.d.	33.0 ± 13.8	u.d.	36.5 ± 8.9
	Nitrite [μM]	1.4 ± 1.3	25.1 ± 8.9^*^	0.5 ± 1.4	21.2 ± 6.6
		Control	LPS		LPS + 1 μM MRS
	% Microglia	10.0 ± 0.9	22.6 ± 0.7^*^^*^^*^		18.3 ± 0.3
	TNFα [pg/mL]	u.d.	560.0 ± 41.7^*^^*^^*^		343.4 ± 31.1
	IL-1β [pg/mL]	2.7 ± 0.4	27.0 ± 4.7^*^^*^		36.7 ± 2.2
	Nitrite [μM]	0.8 ± 0.1	3.7 ± 0.6^*^^*^		3.1 ± 0.3
MRS2578 7 days		Control	LTA		LTA + 1 μM MRS
	% Microglia	8.5 ± 0.8	41.5 ± 3.4^*^^*^^*^		34.9 ± 3.2
		Control	LPS		LPS + 1 μM MRS
	% Microglia	9.7 ± 1.2	28.4 ± 1.3^*^^*^^*^		23.3 ± 2.3

B. Reactive oxygen and nitrogen species releasing reagents				
Condition	Measure	Experimental conditions		
Medium (no cells)	Nitrite [μM]	Control	5 μM ONOO−	50 μM SIN-1
		u.d.	6.3 ± 0.3	18.2 ± 0.01
Mixed cultures		Control	5 μM ONOO−	ONOO− + 1 μM MRS2578
	% Microglia	4.2 ± 0.9	5.6 ± 1.6	4.9 ± 1.1
	Nitrite [μM]	1.6 ± 0.07	7.1 ± 1.5^*^	6.7 ± 1.2
	TNFα	u.d.	u.d.	u.d.
		Control	50 μM SIN-1	SIN-1 + 1 μM MRS2578
	% Microglia	3.7 ± 0.8	3.9 ± 0.4	3.5 ± 0.2
	Nitrite [μM]	2.0 ± 0.2	21.4 ± 2.1	23.6 ± 1.1
	TNFα	u.d.	u.d.	u.d.

A: Microglial inflammation was measured for the experiments described in the main text. “u.d.” indicates that the inflammatory mediator was undectectable. Unless otherwise specified, measurements were performed at 3 days after stimulation with lipoteichoic acid (LTA, 50 μg/mL) or lipopolysaccharide (LPS, 100 ng/mL). Microglial densities are given as % of the neuronal number in control cultures. Nitric oxide release was measured by its oxidation product nitrite. B: The breakdown of peroxynitrite and SIN-1 (which produces peroxynitrite) is measured through the production of nitrite. Data shown are means ± s.e.m. for at least three independent experiments. “^*^” and “#” indicate comparison with control and LTA/LPS, respectively, ^*^/^*^^*^/^*^^*^^*^.

To confirm that purinergic P2X/P2Y receptors were involved in the inflammatory neuronal loss, cultures were treated with the nonspecific P2X/P2Y receptor antagonist RB2 (Davalos et al., 2005; Glänzel et al., 2002). RB2 (100 μM) completely prevented neuronal loss for up to 7 days after LTA or LPS stimulation (at 10 μM, RB2 only partially blocked neuronal loss; Fig. 1), without preventing LTA- and LPS-induced microglial proliferation (Table1). These results indicate an essential role of P2X/P2Y purinergic signaling to neuronal loss under inflammatory conditions.

**Figure 2:**
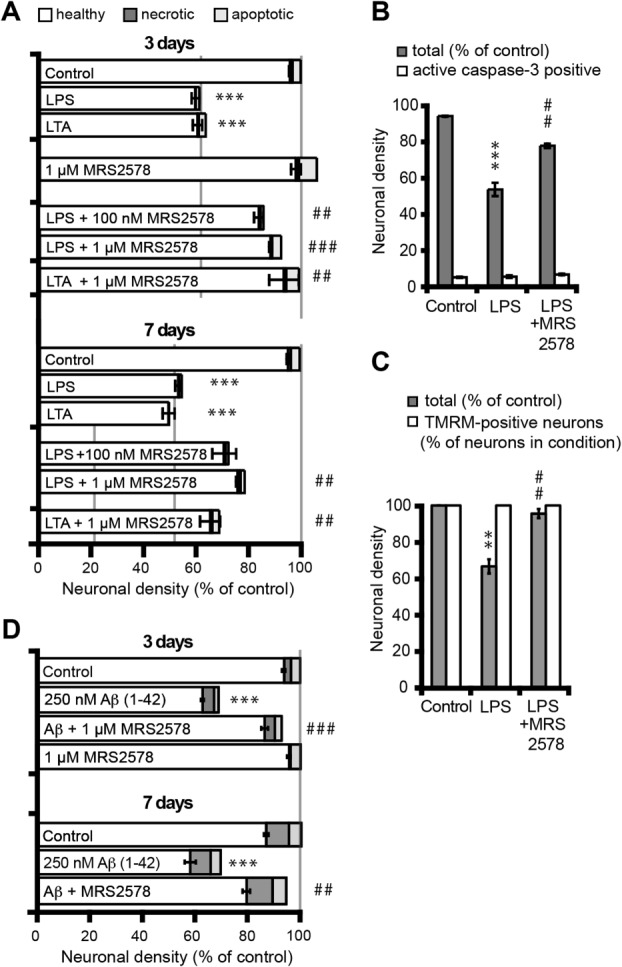
Specific inhibition of the P2Y_6_ receptor prevents inflammatory neurodegeneration induced by LTA, LPS, and amyloid-β (1–42). A: Daily treatment of cultures with the P2Y_6_ receptor antagonist MRS2578 (MRS, 1 μM) inhibits neuronal loss, as evaluated by neuronal densities at 3 and 7 days after lipoteichoic acid (LTA, 50 μg/mL) or lipopolysaccharide (LPS, 100 ng/mL) stimulation. Note that microglial densities and the production of TNF-α, IL-1β, and nitrite (a breakdown product of nitric oxide) are increased in response to LTA/LPS and are not affected by treatment with MRS2578 (see Table1). B: Densities of healthy and active caspase-3 positive neurons at 3 days after LPS (100 ng/mL) stimulation. Protection provided by MRS2578 does not lead to accumulation of caspase-3 positive cells. C: Neurons protected by MRS2578 treatment have normal plasma membrane and mitochondrial membrane potential, as evaluated by tetramethylrhodamine-methyl ester (TMRM) staining. D: Daily treatment of cultures with the P2Y_6_ receptor antagonist MRS2578 (MRS, 1 μM) inhibits neuronal loss, as evaluated by neuronal densities at 3 and 7 days after amyloid-β (1–42) treatment. Data are presented as means ± s.e.m. for at least three independent experiments; */**/***P < 0.05/0.01/0.001 versus control, #/##/###*P* < 0.05/0.01/0.001 versus LTA, LPS, or amyloid-β.

Having established a contribution of purinergic signaling to inflammation-mediated neuronal loss, the specific participation of the microglial P2Y_6_ receptor was investigated. This receptor has been directly linked to microglial engulfment of neurons (Koizumi et al., 2007), but the outcome of inhibiting this signaling on neuronal survival has not been analyzed. The P2Y_6_-specific irreversible antagonist MRS2578 (Mamedova et al., 2004) was added daily to LTA/LPS-stimulated cultures. Blocking P2Y_6_ activation with 1 μM MRS2578 resulted in marked neuroprotection at 3 days after LTA/LPS stimulation, while a lower concentration (100 nM) showed only partial protection; at 7 days, the increase in apparently healthy neurons was less pronounced, but still very significant (Fig. 2A). The neurons saved from LPS-induced neuronal loss did not have active caspase-3 (Fig. 2B) and had intact mitochondrial and plasma membrane potentials as demonstrated by TMRM staining (Nicholls, 2006; Fig. 2C), indicating that they were healthy. Importantly, MRS2578 did not inhibit the LTA- or LPS-induced microglial proliferation or the release of inflammatory mediators TNF-α, IL-1β and NO (Table1), suggesting that the survival of neurons was not due to a reduced inflammatory response. These findings indicate that P2Y_6_ receptor signaling is crucial for the induction of neuronal death through microglial phagocytosis during LPS- or LTA-induced inflammation.

### P2Y_6_ Receptor Signaling Is Required for Neuronal Loss Induced by Various Neuroinflammatory Stimuli

Microglial phagocytosis-dependent neuronal loss can be induced by a variety of stimuli other than TLR agonists, such as sub-neurotoxic concentrations of the Alzheimer's disease-related peptide Aβ_1–42_ (Neniskyte and Brown, 2013; Neniskyte et al., 2011). To assess whether P2Y_6_ receptor signaling is required for neuronal loss induced by Aβ_1–42_, the P2Y_6_ receptor inhibitor MRS2578 was added daily to the mixed neuronal/glial cultures stimulated with Aβ_1–42_ (250 nM). Inhibition of P2Y_6_ receptor activation prevented neuronal loss for up to 7 days (Fig. 2D), confirming that P2Y_6_ receptor inhibition did not merely delay neuronal death.

**Figure 3:**
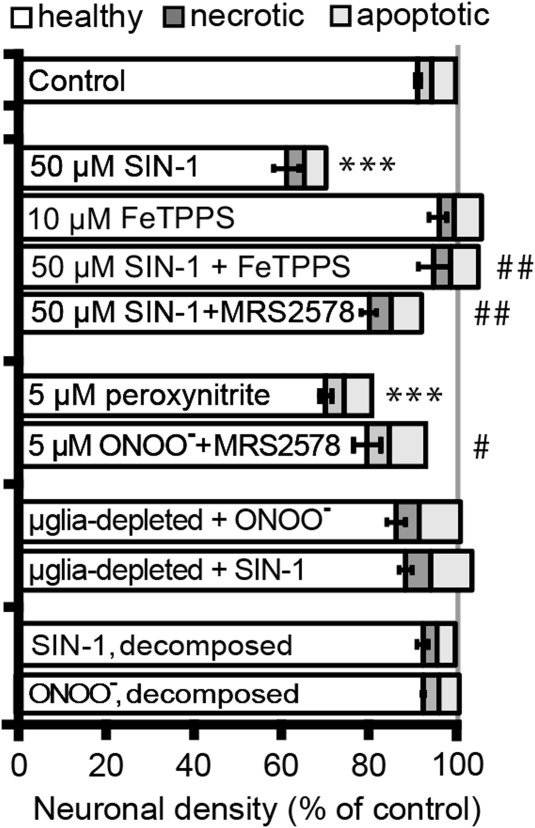
Inhibition of P2Y_6_ receptors prevents neuronal loss induced by peroxynitrite. Mixed neuronal/glial cultures were treated with peroxynitrite (5 μM), or a peroxynitrite donor SIN-1 (50 μM) for 3 days with or without the selective P2Y_6_ inhibitor MRS2578 (added every day). MRS2578 provides partial protection against neuronal loss induced by peroxynitrite or SIN-1. Neuronal loss induced by SIN-1 is also prevented by a peroxynitrite scavenger FeTTPS, and neuronal loss induced by SIN-1 or peroxynitrite is prevented by depleting microglia from the cultures using l-leucine-methyl ester (LME). Note that MRS2578 did not affect microglial numbers or the production of reactive oxygen species by peroxynitrite or SIN-1 (see Table1). Data are presented as means ± s.e.m. for at least three independent experiments; **/***P < 0.01/0.001 versus control, #/##/###*P* < 0.05/0.01/0.001 versus peroxynitrite or SIN-1.

We have previously shown that LPS-, LTA-, and Aβ_1–42_-induced neuronal loss is mediated by peroxynitrite release from microglia and that peroxynitrite itself can induce neuronal phagoptosis (Neher et al., 2011b). Therefore, we tested whether the neuronal loss induced by 5 μM peroxynitrite or 50 μM of the peroxynitrite-generating agent SIN-1 could be blocked by MRS2578. The neuronal loss induced by SIN-1 was prevented by a peroxynitrite scavenger FeTTPS (Fig. 3) indicating it was indeed mediated by peroxynitrite, and neuronal loss induced by SIN-1 or peroxynitrite was prevented by eliminating microglia from the cultures (0.7 ± 0.2% microglia; Fig. 3) showing that it was mediated by microglia. Importantly, we found that MRS2578 did indeed inhibit the neuronal loss induced by peroxynitrite or SIN-1 (Fig. 3) demonstrating that this neuronal loss was mediated by P2Y_6_ receptors. Importantly, MRS2578 did not affect the levels of peroxynitrite as measured by the levels of its breakdown product nitrite (Table1), indicating that it was acting on the P2Y_6_ receptor rather than reacting with the reagents.

Note that in all cases (LPS, LTA, Aβ, peroxynitrite, and SIN-1), blocking microglial phagocytosis of neurons with MRS2578 prevented neuronal loss without increasing the number of apoptotic or necrotic neurons (Figs. 2 and 3) or affecting microglial numbers (Table1), indicating that phagocytosis was the cause of death, rather than its consequence. If microglia had killed the neurons first and subsequently phagocytosed them, then inhibition of phagocytosis would have left dead not live neurons.

### Stimulation of the P2Y_6_ Receptor with UDP Induces Microglia-Dependent Neuronal Loss

The above work indicates that activation of the P2Y_6_ receptor is required for inflammatory neuronal loss, but does not show whether activation of the P2Y_6_ receptor alone is sufficient to cause neuronal loss by phagoptosis.

**Figure 4:**
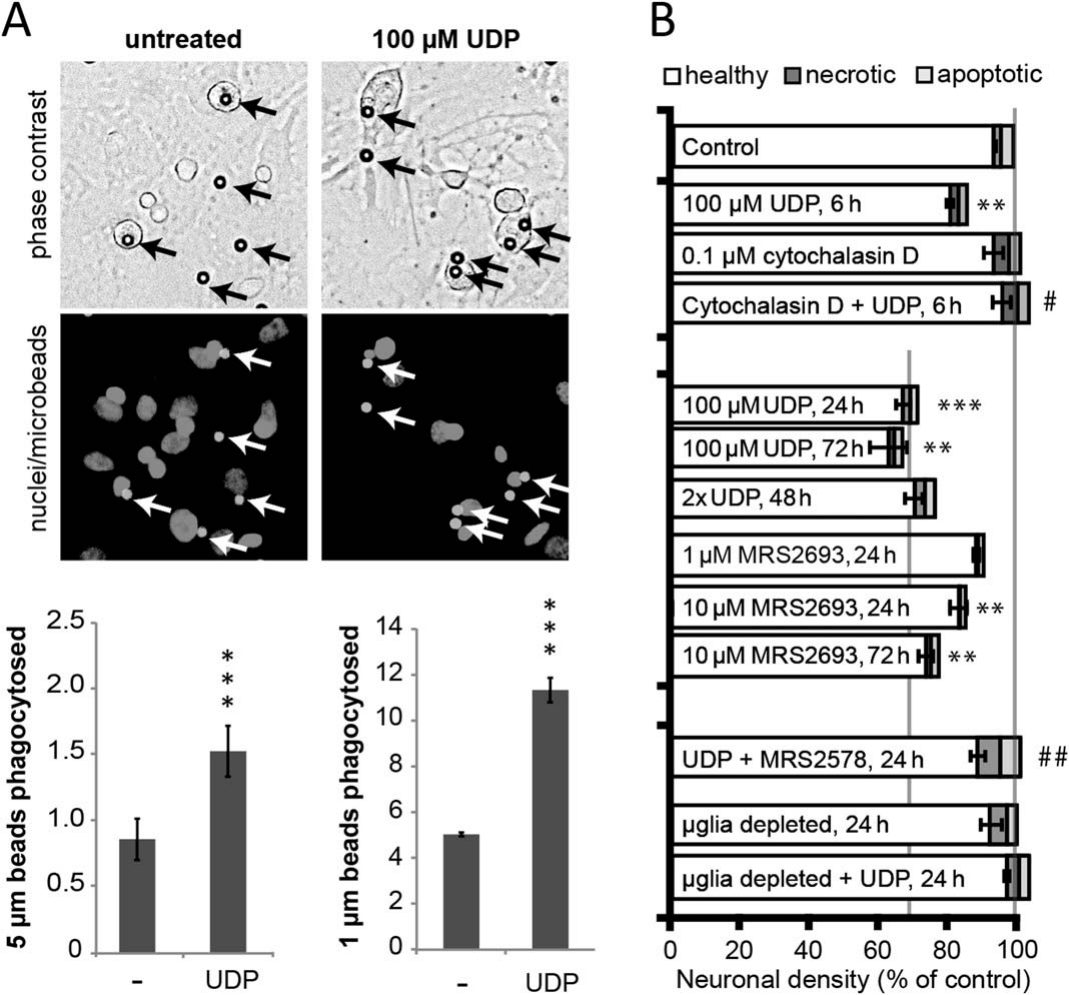
Exogenous UDP induces rapid, microglia-dependent neuronal loss through P2Y_6_ receptor activation. A: Microglia in mixed glial cultures treated acutely with 100 µM UDP show enhanced phagocytosis of fluorescent microbeads (micrographs show phagocytosis of 5 µm large microbeads; arrows). B: In mixed neuronal/glial cultures, the P2Y_6_ agonist UDP (100 μM) induces neuronal loss within 6 h, and this neuronal loss is blocked by co-application of the general phagocytosis inhibitor Cytochalasin D (100 nM). This UDP-induced neuronal loss is greater at 24 h, but is neither enhanced by the application of a second dose of UDP after an additional 24 h nor does it progress significantly further at 72 h. The synthetic P2Y_6_ agonist, MRS2693 induces neuronal loss equivalent to UDP. Importantly, UDP-induced neuronal loss is prevented by microglial depletion (with l-leucine methyl ester) or application of the selective P2Y_6_ inhibitor MRS2578 (MRS, 1 μM). Note that neither UDP nor MRS2693 affected microglial proliferation in any of the conditions (not shown). Data are presented as means ± s.e.m. for at least three independent experiments; */**/***P < 0.05/0.01/0.001 versus control, ##/###*P* < 0.01/0.001 versus UDP.

The endogenous agonist for P2Y_6_ receptors is thought to be UDP (Chang et al., 1995). Exogenous UDP (100 μM) has previously been reported to promote microglial phagocytosis via P2Y_6_ activation (Koizumi et al., 2007) and we confirmed here that acute treatment of mixed glial cultures with 100 μM UDP strongly enhanced microglial phagocytic activity as measured by their uptake of microbeads (1 or 5 μm diameter and negatively charged, Fig. 4A).

Interestingly, 100 μM UDP applied to mixed neuronal/glial cultures caused rapid loss of healthy neurons within 24 hours of addition (Fig. 4B) and a second dose of UDP, added at the same concentration 24 h after the first one, did not cause further neuronal loss (Fig. 4B). Furthermore, neuronal loss did not increase after incubation of cultures for 72 h (Fig. 4B), indicating that UDP-induced neuronal loss was rapid and nonprogressive, in line with a short-term signal for phagocytosis. In fact, we found that 100 μM UDP for just 6 hours induced neuronal loss that was less pronounced than after 24 hours but still very significant, and without increasing apparent apoptosis or necrosis (Fig. 4B). In order to test whether phagocytosis was required for this UDP-induced neuronal loss, an inhibitor of actin polymerization and therefore phagocytosis, cytochalasin D, was added at the same time as UDP. Cytochalasin D completely prevented the UDP-induced neuronal loss, leaving live neurons, indicating that phagoptosis was the likely cause of neuronal death induced by UDP (Fig. 4B).

To test if UDP induced phagocytosis through activation of the P2Y_6_ receptor, we applied the specific, irreversible P2Y_6_ inhibitor MRS2578. Neuronal loss induced by UDP was prevented by inhibiting P2Y_6_ receptors with MRS2578 (Fig. 4B), indicating that UDP-induced neuronal loss was mediated by activation of P2Y_6_ receptors.

To confirm that activation of P2Y_6_ receptors was sufficient for neuronal loss, we applied the synthetic P2Y_6_ receptor agonist MRS2693 (Besada et al., 2006) to neuronal-glial cultures, and found that equivalent to UDP this induced significant neuronal loss without increasing neuronal necrosis or apoptosis (Fig. 4B). These results indicate that activation of the P2Y_6_ receptor alone is sufficient to induce phagoptosis.

Finally, in order to test whether microglia were required for UDP-induced neuronal loss, microglia were depleted from the glial-neuronal co-cultures with LME, and this was found to prevent subsequent UDP-induced neuronal loss, leaving live neurons (Fig. 4B). This demonstrates that microglia are required for UDP-induced loss of neurons and that UDP is not directly toxic to neurons.

### LPS-Induced Neuronal Loss *In Vivo* Is Prevented by Inhibition of P2Y_6_ Signaling

**Figure 5:**
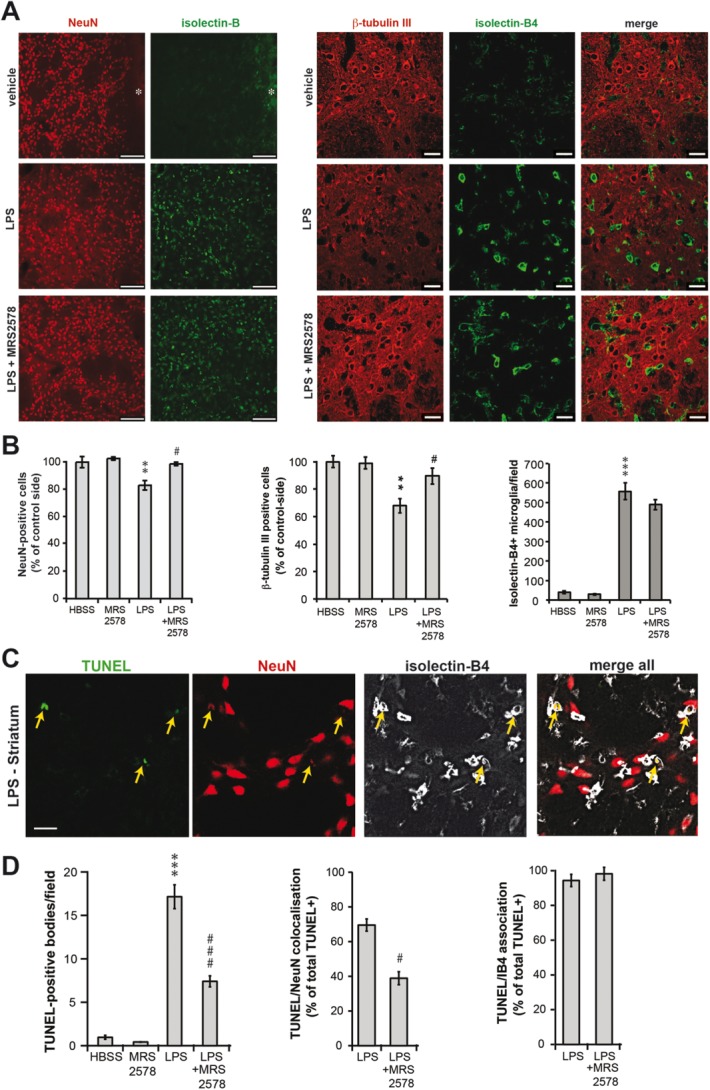
Inhibition of the P2Y_6_ receptor by MRS2578 prevents LPS-induced inflammatory neurodegeneration *in vivo* by preventing microglial phagocytosis of neurons. A: Confocal micrographs of coronal sections through the rat striatum stained for neuronal nuclei (NeuN), neuronal cytoskeleton (β-tubulin III) and activated microglia (isolectin-B_4_). Control injection of Hank's buffered salt solution (HBSS) only results in microglial activation close to the injection site (asterisk), whereas injection of LPS (5 μg) leads to widespread inflammation. B: Injection of LPS alone (*n* = 7) results in neuronal loss (relative to the contralateral striatum), whereas co-injection of the P2Y_6_ receptor inhibitor MRS2578 (100 μM, *n* = 5) prevents neuronal loss. LPS-injection also leads to strong microglial activation, which is not inhibited by MRS2578 treatment. C: Small TUNEL-positive inclusions are found throughout the striatum after LPS injection, co-localize with neuronal nuclear staining (NeuN) and are surrounded by microglial membranes, indicative of neuronal phagocytosis by microglia. D: The number of TUNEL-positive nuclei is strongly reduced by co-injection of MRS2578 (100 µM), indicating that blocking the P2Y_6_ receptor prevents neuronal phagocytosis and death. Scale bar 100 μm (A, left) or 20 μm (A, right and C). Data are presented as means ± s.e.m. for five brain sections and four fields per section; **/***P < 0.01/0.001 versus HBSS, #*P* < 0.05 versus LPS. [Color figure can be viewed in the online issue, which is available at wileyonlinelibrary.com.]

We have previously reported that LPS injection into the striatum causes inflammatory neuronal loss that can be prevented by inhibiting the PS/MFG-E8/VNR phagocytic pathway of microglia (Fricker et al., 2012a). To test whether P2Y_6_ signaling is also required for microglial phagocytosis of neurons *in vivo*, rats were injected into the striatum with LPS (5 μg) with or without the P2Y_6_ receptor antagonist MRS2578 (100 μM). Co-injection with MRS2578 significantly prevented loss of neurons at 3 days after LPS injection, as evaluated by neuron-specific NeuN or β-tubulin III immunoreactivity (Fig. 5A,B).

As expected, LPS-injection induced activation of microglia as detected by their affinity for isolectin-B4 (Maddox et al., 1982; Zhang et al., 1997). Importantly, the number of isolectin-positive microglia was not reduced by co-treatment with MRS2578 (Fig. 5B), confirming that the protective effects observed were not due to reduced microglial activation, in line with our *in vitro* results. Also, blocking microglial phagocytosis of neurons with MRS2578 did not increase the number of dying neurons as demonstrated by the absence of neurons staining for activated caspase-3 and Fluorojade C, which were only found around the injection site (Supp. Info. Fig. 1). However, we have previously found that neuronal nuclei become TUNEL positive (i.e., have DNA double strand breaks) after phagocytosis by microglia *in vivo* (Fricker et al., 2012a). Accordingly, there was a strong increase in the number of small TUNEL-positive/NeuN-positive inclusions after LPS-injection. Importantly, the number of TUNEL stained-neuronal nuclei found surrounded by microglial membrane were strongly reduced by cotreatment with MRS2578 (Fig. 5C,D), indicating that phagocytosis of neurons and their degradation inside of microglia was prevented by inhibition of the P2Y_6_ receptor. Thus, inhibition of P2Y_6_ phagocytic signaling is sufficient to block phagocytosis of stressed but viable neurons and thereby prevents inflammatory neuronal loss and death *in vivo*.

## Discussion

We have previously shown that the TLR agonists LPS, LTA, and Aβ induce delayed neuronal loss in glial-neuronal co-cultures that is prevented by: (i) eliminating microglia, (ii) separating microglia from neurons by a permeable transwell membrane, (iii) inhibiting peroxynitrite production, (iv) blocking PS exposed on neurons with PS-binding proteins, (v) inactivating or knocking out the PS-binding protein MFG-E8, (vi) blocking the VNR that acts as a phagocytic receptor for PS-bound MFG-E8, (vii) blocking calreticulin exposed on neurons, or (viii) blocking LRP, which acts as phagocytic receptor for calreticulin (Fricker et al., [Bibr b12],[Bibr b13]; Neher et al., [Bibr b28],[Bibr b26]; Neniskyte and Brown, 2013; Neniskyte et al., 2011). Neuronal loss is accompanied by microglial phagocytosis of neurons as indicated by video-imaging and the accumulation of neuronal nuclei within microglia (Fricker et al., 2012a; Neher et al., 2011b). Inhibition of phagocytosis by the above methods prevents neuronal loss and the saved neurons are viable, indicating that the neurons phagocytosed were otherwise viable, rather than being phagocytosed after death, when inhibition of phagocytosis would result in the accumulation of dead neurons.

Inflammatory-activated microglia produce NO from iNOS and superoxide from the phagocyte NADPH oxidase, resulting in the production of extracellular peroxynitrite (Bal-Price et al., 2002), which induces reversible PS-exposure on neurons (Neher et al., 2011b). This exposed PS is bound by MFG-E8 produced by activated glia, which stimulates phagocytosis of the PS-exposed neurons via the phagocytic VNR (Fricker et al., 2012a; Neher et al., 2011b). This microglial phagocytosis of otherwise viable neurons might contribute to the inflammatory neuronal loss that occurs in neurological diseases such as meningitis, encephalitis, trauma, and Alzheimer's disease (Brown and Neher, 2014; Neher et al., 2012) and we have recently shown that it mediates delayed neuronal loss after focal brain ischemia (Neher et al., 2013). Others have shown that primary phagocytosis of otherwise-viable neurons by activated microglia mediates neuronal loss and death in models of AIDS dementia, Parkinson's disease, Motor Neuron Disease, and normal developmental loss of neuronal precursors (Barcia et al., 2012; Cunningham et al., 2013; Liu et al., 2012; Marker et al., 2012). Thus, it is important to understand the signaling between neurons and microglia that drives phagoptosis, and thereby devise therapies that prevent it in neurological diseases.

Koizumi et al. (2007) found that: (i) microglia expressed P2Y_6_ receptors, and this expression was upregulated by kainate treatment of rats, (ii) UDP activated microglia via P2Y_6_ receptors to phagocytose zymosan and polystyrene beads, (iii) kainate-treated neurons released UTP, and (iv) kainate-treated rats had seizures and 2–3 days later lost hippocampal neurons in association with activated microglia, UTP release, and increased microglial phagocytosis of beads infused into the brain that was blocked by knockdown or inhibition of P2Y_6_ receptors. Koizumi et al. (2007) concluded that UDP/UTP release from damaged neurons induces local microglia to phagocytose those neurons via activating microglial P2Y_6_ receptors. Koizumi et al. (2007) did not speculate or investigate whether such phagocytosis might contribute to the neuronal death caused by the kainate-induced seizures, but this is clearly a possibility that merits testing.

We found that UDP alone was sufficient to induce neuronal loss mediated by microglia. In contrast to the phagocytic neuronal loss induced by LPS, LTA, and Aβ, which occurs after a delay of 2 days (Neher et al., 2011b; Neniskyte et al., 2011), the neuronal loss induced by UDP was rapid (Fig. 4). This is consistent with UDP activating formation of the phagocytic cup (Koizumi et al., 2007). Thus, UDP is an “eat-me” signal, and, although soluble, may be spatially localized by rapid breakdown by ectonucleases (Koizumi et al., 2007). UDP-evoked phagocytosis of neurons may function to remove dying or damaged neurons, but there is a clear danger that stressed but functional neurons may be removed and thereby killed as well. The neurons that we saved from phagocytosis induced by LPS, LTA, and Aβ by inhibiting the P2Y_6_ receptors were viable for at least 7 days and had normal plasma and mitochondrial membrane potentials, suggesting that they were functional (Fig. 2) before being phagocytosed. Importantly, the rapid loss of neurons in response to UDP treatment suggests that as the final step in the phagocytic uptake, UDP may induce bystander uptake of neurons without a requirement for other eat-me signals and ignoring don't-eat-me signals. However, this requires further investigation.

Furthermore, we found that *in vivo* LPS caused the loss of striatal neurons, which was prevented by co-injection of the P2Y_6_ receptor inhibitor MRS2578. LPS injection also led to the appearance of small TUNEL-positive inclusion bodies, which co-stained for the neuronal nuclear marker NeuN. Importantly, these neuronal particles were virtually always surrounded by microglial membrane, indicative of phagocytic uptake. Strikingly, the occurrence of these inclusions was strongly reduced by the P2Y_6_ inhibitor MRS2578, indicating that neuronal nuclear fragmentation was only induced after phagocytic uptake. In line with this interpretation, the density of neurons was higher in LPS + MRS2578 injected animals, while no accumulation of dying neurons could be observed (based on staining for Fluorojade C and active caspase-3). This suggests that neuronal death was induced through the phagocytic process and that UDP/P2Y_6_ signaling is a crucial mediator for neuronal uptake and death during inflammation. Thus, preventing UDP/P2Y_6_ mediated phagocytosis of neurons is potentially beneficial, although this needs to be examined with functional tests *in vivo*.

**Figure 6:**
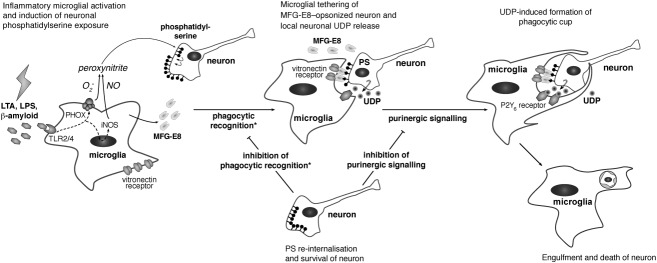
Schematic representation of the molecular mechanism leading to phagoptosis of neurons by microglia during inflammation. We have previously shown (marked by asterisk) that microglial inflammation causes production of nitric oxide (NO) and superoxide (O_2_^−^), forming peroxynitrite and leading to neuronal phosphatidylserine (PS) exposure. Exposed PS is then recognized by the bridging protein MFG-E8, which also binds the microglia vitronectin receptor leading to neuronal “tethering” (Fricker et al., 2012a; Neher et al., [Bibr b28],[Bibr b26]; Neniskyte and Brown, 2013). Here, we show that release of UDP and its activation of the P2Y_6_ receptor is also required for the uptake and death of stressed but otherwise viable neurons.

We found here (summarized in Fig. 6) that the neuronal loss induced by LPS or LTA was prevented by: (i) delayed addition of extracellular apyrase, which degrades nucleoside triphosphates and diphosphates, indicating that extracellular nucleoside triphosphates or diphosphates are required for neuronal loss, (ii) RB2, which nonspecifically inhibits purinergic receptors, indicating that purinergic signaling is required for neuronal loss, and (iii) MRS2578, which specifically blocks P2Y_6_ receptors, indicating that activation of these receptors is required for neuronal loss. Blocking P2Y_6_ receptors with MRS2578 also prevented phagoptosis of neurons induced by Aβ_1–42_ and peroxynitrite (authentic or when produced from the degradation of SIN-1). Furthermore, *in vivo* injection of LPS into rat striatum induced microglial activation and delayed neuronal loss that was prevented by blocking P2Y_6_ receptors with MRS2578. Thus, blocking UDP/P2Y_6_ signaling is sufficient to prevent neuronal loss and death induced by a wide range of stimuli activating microglial phagocytosis of neurons. It will be important to test whether this is beneficial in animal models of neurological disease.

## Abbreviations

Aβ_1–42_: amyloid beta 1–42
HBSS: Hank's balanced salt solution
LPS: lipopolysaccharide
LTA: lipoteichoic acid
PS: phosphatidylserine
RB2: reactive blue 2
SIN-1: 3-morpholinosydnonimine
TNF-α: tumor necrosis factor-α
VNR: vitronectin receptor
