# Supplementary figures and images for: Inhibition of UDP/P2Y6 purinergic signaling prevents phagocytosis of viable neurons by activated microglia in vitro and in vivo
Source: Glia. 2014 May 19;62(9):1463–75. doi: 10.1002/glia.22693 (PMC4336556; doi:10.1002/glia.22693)

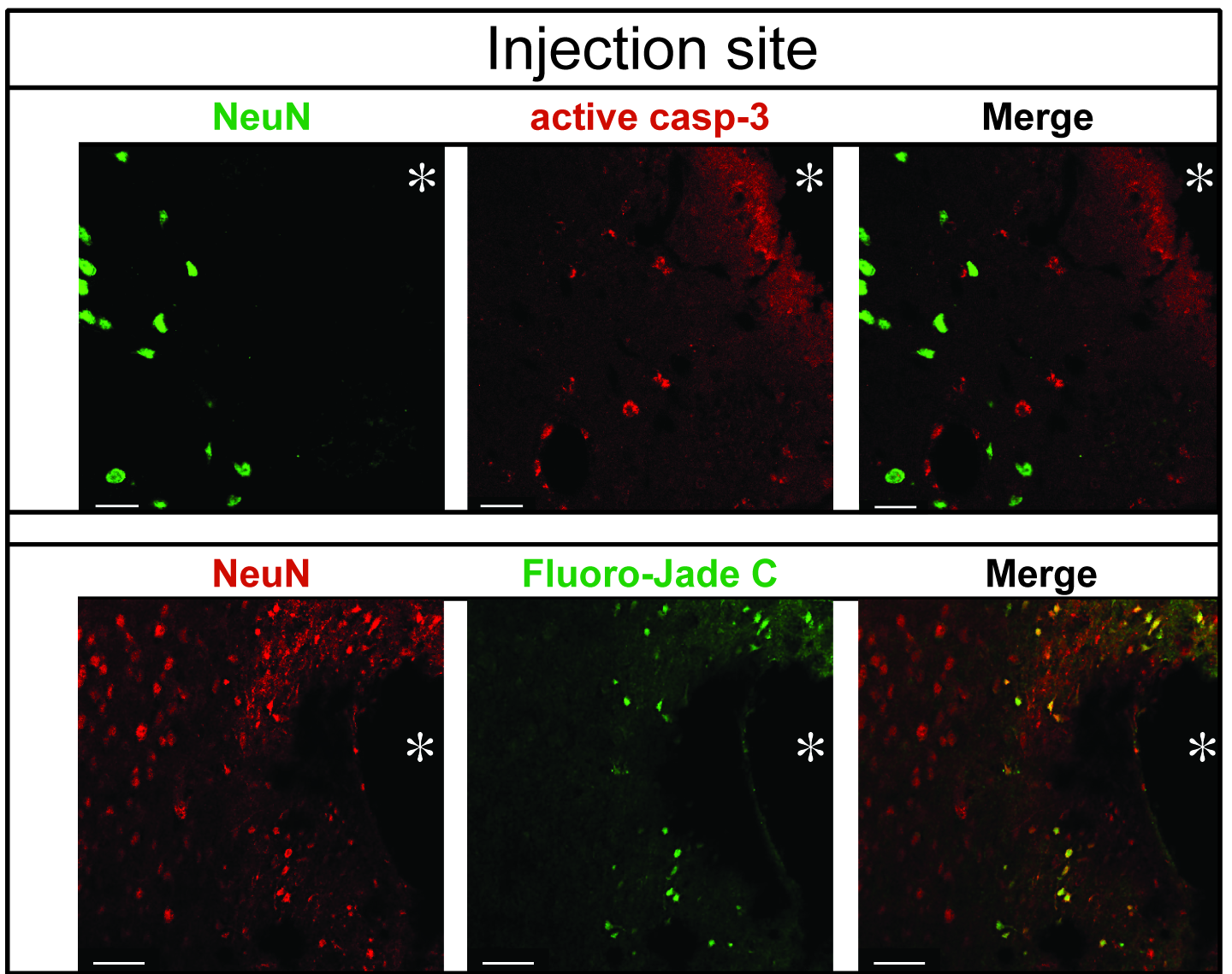

Supplement: Supplementary file 1 — Figure 1 [file glia0062-1463-sd1.tif]
